# LRpath analysis reveals common pathways dysregulated via DNA methylation across cancer types

**DOI:** 10.1186/1471-2164-13-526

**Published:** 2012-10-04

**Authors:** Jung H Kim, Alla Karnovsky, Vasudeva Mahavisno, Terry Weymouth, Manjusha Pande, Dana C Dolinoy, Laura S Rozek, Maureen A Sartor

**Affiliations:** 1 Department of Computational Medicine and Bioinformatics, Medical School, University of Michigan, Ann Arbor, MI, USA; 2 Department of Environmental Health Sciences, School of Public Health, University of Michigan, Ann Arbor, MI, USA; 3 Neurology Department, Medical School, University of Michigan, Ann Arbor, MI, USA; 4 Department of Otolaryngology, Medical School, University of Michigan, Ann Arbor, Mi, USA

## Abstract

**Background:**

The relative contribution of epigenetic mechanisms to carcinogenesis is not well understood, including the extent to which epigenetic dysregulation and somatic mutations target similar genes and pathways. We hypothesize that during carcinogenesis, certain pathways or biological gene sets are commonly dysregulated via DNA methylation across cancer types. The ability of our logistic regression-based gene set enrichment method to implicate important biological pathways in high-throughput data is well established.

**Results:**

We developed a web-based gene set enrichment application called LRpath with clustering functionality that allows for identification and comparison of pathway signatures across multiple studies. Here, we employed LRpath analysis to unravel the commonly altered pathways and other gene sets across ten cancer studies employing DNA methylation data profiled with the Illumina HumanMethylation27 BeadChip. We observed a surprising level of concordance in differential methylation across multiple cancer types. For example, among commonly hypomethylated groups, we identified immune-related functions, peptidase activity, and epidermis/keratinocyte development and differentiation. Commonly hypermethylated groups included homeobox and other DNA-binding genes, nervous system and embryonic development, and voltage-gated potassium channels. For many gene sets, we observed significant overlap in the specific subset of differentially methylated genes. Interestingly, fewer DNA repair genes were differentially methylated than expected by chance.

**Conclusions:**

Clustering analysis performed with LRpath revealed tightly clustered concepts enriched for differential methylation. Several well-known cancer-related pathways were significantly affected, while others were depleted in differential methylation. We conclude that DNA methylation changes in cancer tend to target a subset of the known cancer pathways affected by genetic aberrations.

## Background

Since the introduction of the Illumina HumanMethylation27 BeadChip platform, which measures the methylation of over 27,000 CpG sites across the human genome, several studies have reported genomic sites with aberrant methylation in cancers. These publicly available datasets, including several performed by The Cancer Genome Atlas (TCGA), now allow for an integrative analysis of DNA methylation across multiple cancer types. We took a pathway-level approach to this integrative analysis, illustrating the use of our newly developed gene set enrichment testing web-based application, LRpath ( http://lrpath.ncibi.org).

The identification of predefined sets of biologically related genes enriched with differentially expressed genes is used routinely in the analysis and interpretation of data from microarrays, RNA-Seq, and other high-throughput methods. The most commonly used approach to identifying enriched sets of genes is based on counting the number of differentially expressed genes in a particular biological concept. A biological concept is a pre-defined, biologically-related set of genes, derived from any one of a number of different annotation sources [1]. In particular, such focus on biological concepts rather than individual genes has proven useful in cancer research. Several groups have developed tools looking at the change in groups of genes sharing the same functions or regulatory modules, as detailed in Furney et al., where additional resources for cancer genomic and epigenomic studies can be found [2]. Enrichment analysis is not limited to transcriptomic data; pathway analysis using epigenetic changes can also provide valuable information as demonstrated by a lymphoma study where inflammatory signalling, especially the tumor necrosis factor α network, was found to be differently dysregulated between two tumor subtypes [3]. For the analyses conducted in this manuscript, we used genes harbouring differentially methylated CpG sites in their promoter proximity, rather than differential expression, in multiple cancer types. The statistical significance of such overlap between genes of interest and a particular concept is often established using Fisher’s exact test. A number of tools that utilize this, or a very similar approach have been developed, such as David/EASE [4,5], Onto-Express [6,7], ConceptGen [1], the Gostats package of Bioconductor [8], GOMiner [9,10], and FuncAssociate [11].

As all of these programs require a list of differentially expressed genes as input, the analytical results are influenced by the significance cut-off selected by the user. Thus, several methods have been proposed that offer alternative approaches that do not require a significance cut-off. Gene Set Enrichment Analysis (GSEA) uses differential expression statistics of all genes, without categorizing them into differentially and non-differentially expressed, and a non-parametric method to identify enriched gene sets [12]. Our recently published LRpath method uses logistic regression to functionally relate the odds of gene set membership with the significance of differential expression and calculates adjusted P-values as a measure of statistical significance [13]. An alternative interpretation of how LRpath works comes from the random sets method; that is, LRpath tests whether the significance levels of a particular set of genes is significantly higher (or lower) than those of a randomly chosen set of genes of the same size [13,14].

We recently developed a web-based application for LRpath with greatly expanded and novel gene set annotations, including metabolite, transcription factor and microRNA target sets, and literature-derived annotations, and that also includes clustering analysis functionality, allowing one to identify and compare biological concept signatures across multiple studies. LRpath is particularly suitable for such an integrative study, because it performs well with both small and large sample sizes [13], as it does not depend on non-parametric resampling of samples to assess significance of enrichment. Additional benefits of using the LRpath program include (1) the ability to perform both “directional” and “non-directional” enrichment tests that allow for two different perspectives to enhance interpretation and (2) the ability to easily compare and visualize results across multiple studies using LRpath clustering functionality.

Epigenetic mechanisms such as DNA methylation and histone modifications play essential roles in cell differentiation and transcriptional regulation and are identified as key mediators of cancer progression. For example, transcription of a number of tumor suppressor genes such as *p16*^*INK4a*^, *BRCA1*, *p53* and *MLH1* has been demonstrated to be silenced by promoter hypermethylation [15]. Furthermore, genomic instability associated with the hypermethylation of the DNA mismatch repair enzyme gene *MLH1* may not only deregulate critical genes involved in the initial stages of carcinogenesis, but also those involved in the later invasion and metastasis stages of transformation [16].

In cancer, recurrent patterns of aberrant DNA methylation alteration are evident, especially in promoter regions, implicating the contribution of specific altered pathways driven by methylation change. For example, DNA hypermethylation of gene promoters commonly marks disease progression and silencing of putative tumor suppressor genes. Conversely, DNA hypomethylation occurs most commonly in a genome-wide manner, especially within repeat elements such as LINE1, Alu, and PG4s (potentially G-quadruplex-forming sequences) [17-19] and is associated with genomic instability [20,21]. Recently, the hypomethylation of PG4-dense regions were reported in cancer, indicating the role of DNA methylation in genomic stability through a structural change in G4 formation, resulting in DNA breakpoint hotspots [19]. In general, demethylation of the genome can lead to 1) the reactivation of transposable elements, thereby altering the transcription of adjacent genes, 2) the activation of oncogenes such as *H-RAS,* and 3) the biallelic expression of imprinted loci (e.g. loss of *IGF2* imprinting) [22-24]. Studies of aberrant DNA methylation can benefit diagnostic and prognostic marker discovery by identifying frequent methylation targets and also can provide new insights for improved classification, diagnosis, therapies, and prognosis.

The relative contribution of epigenetic mechanisms to multiple cancer types is not well understood, in particular to what extent epigenetic mechanisms target similar genes and pathways as somatic mutations. Here, we hypothesize that during the pathogenesis of cancer, certain pathways or biological gene groups are commonly dysregulated via DNA methylation across cancer types. To test our hypothesis, we employed LRpath and clustering analysis on data from ten tumor versus normal DNA methylation studies to unravel the commonly altered pathways and other biological concepts across multiple cancers. The ability of the method employed by LRpath to implicate important biological pathways and groupings has previously been demonstrated [13]. In this paper, we describe the first example of pathway analysis coupled with the DNA methylome of various tumor types.

## Results

### Use of LRpath for enrichment testing and cross-experiment visualization

**Figure 1:**
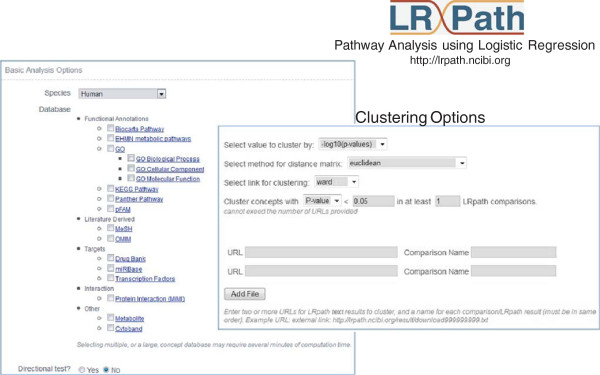
**The user-friendly web interface of LRpath application with 16 different annotation databases with hierarchical clustering functionality.** The first section allows the users to upload the data and select the concepts of interest. The second part of the application allows the users to perform integrative analysis of multiple LRpath results.

The LRpath web application provides a user-friendly web interface, the choice of 16 different annotation databases (see *Methods* for details), and enables visualization of the results of multiple enrichment tests. The first section of the interface allows users to select an organism, upload the input data set, select one or more annotation databases to test against, and set a number of additional parameters (Figure 1 and *Methods*). By default an *undirectional* test is performed, which allows the user to distinguish between ‘Enriched’ concepts (those with more genes changed than expected by chance) and ‘Depleted’ concepts (those with fewer changed). If the user chooses to perform a *directional* test, the concepts enriched with genes that are up- and down-regulated are distinguished, rather than enriched and depleted.

The second part of the application, Cluster Analysis, allows users to integrate LRpath results from multiple experiments in order to interactively view and explore the enrichment profiles across experiments. It provides a user-friendly method for filtering, merging, and clustering LRpath results using several approaches (see *Methods*).

### Identification of biological concepts whose genes tend to be hyper- or hypo- methylated across cancer types (Directional LRpath analysis)

**Figure 2:**
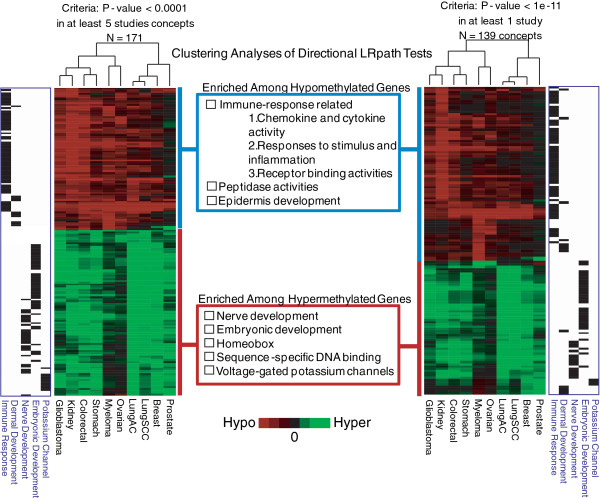
**Hierarchical clustering of significant biological concepts from directional LRpath tests.** The data from 10 different cancer versus normal CpG methylation studies were subjected to directional LRpath analysis and then clustered using criteria indicated in the figure. The clustering of LRpath results suggests that similar pathways across multiple cancers are affected by differential DNA methylation. The majority of the enriched hypomethylated concepts were immune-response related. Concepts related to early development are enriched with genes harbouring hypermethylation.

We analyzed data from ten tumor versus normal CpG methylation studies detailed in Table 1, for significant differentially methylated sites. We then performed clustering analysis using the LRpath application by filtering to concepts exhibiting significant enrichment (p-value<0.0001) in at least five (50%) of the ten cancer types. The clustering analysis results for Gene Ontology (GO) terms and pathways revealed tightly clustered hyper- and hypo-methylated concepts, strongly suggesting that similar pathways across multiple cancer types are affected by dysregulation of DNA methylation (Figure 2, and Additional file 1: Table S1). Among the ninety two hypermethylated concepts identified in the directional analysis (Figure 1), over 50% of them are involved in early development and morphogenesis such as neurogenesis (FDR < 1×10^-10^ in 5 tumor types), homeobox (FDR < 1×10^-10^ in 5 tumor types), and embryonic development (FDR < 1×10^-7^ in 5 tumor types) (Additional file 2: Figure S1A). Since hypermethylation in the promoter region of a gene often represses gene expression, these pathways may be subject to transcriptional suppression. Nearly all of the remaining hypermethylated concepts are involved in transcription factor activity (FDR < 5×10^-7^ in 5 tumor types) and voltage-gated potassium channels (FDR < 1.5×10^-4^ in 5 tumor types). Frequently altered genes involved in transcription factor activity in various cancer types include homeobox (*HOX*) genes, paired box (*PAX*) genes, and Wilms tumor suppressor gene (*WT1*). Examination showed that the hypermethylation in these gene clusters, which accounts for the enriched hypermethylated concepts involved in transcription activity, is reassured in the majority of the cancer cases (Additional file 2: Figure S2). Among the 1,517 probes on the Illumina BeadChip annotated to a gene with sequence-specific transcription factor activity, 202 probes have more than 20% average change in methylation in at least three tumor types. Unsupervised clustering and visualization of these genes reveal that they were mostly hypermethylated in tumors (185 hypermethylated vs. 17 hypomethylated probes as seen in Additional file 2: Figure S3). While over 80% of the hypermethylated transcription factor genes are PRC2 targets (158 out of 185 probes corresponding to 86 out of 106 unique genes), very few hypomethylated transcription factor genes were PRC2 targets (5 out of 17 probes corresponding to 1 out of 10 unique genes) (Additional file 2: Figure S3).

**Table 1:** Description of datasets used in the study GEO identifiers indicate the GSE ID for the study

**Source**	**GEO**	**GEO**	**GEO**	**GEO**	**TCGA**	**TCGA**	**TCGA**	**TCGA**	**TCGA**	**TCGA**
	**17648**	**21304**	**22867**	**26126**						
Tumor Type	Colon	Multiple Myeloma	Glioblastoma	Prostate	Breast	Kidney	Lung AC	Lung SCC	Ovarian	Stomach
Normal Sample #	22	3	4	71	27	199	24	27	8	57
Cancer Sample #	22	161	77	71	67	199	24	27	39	57
P-value < 0.01	7922	4489	1403	9151	6672	10664	6419	6738	4376	8436
P-value < 0.01 and at least 10% change in average methylation	5642	4343	1179	3263	3888	2022	3847	3641	1900	3000
Matched	Yes	No	No	Yes	No	Yes	Yes	Yes	No	Yes

**Figure 3:**
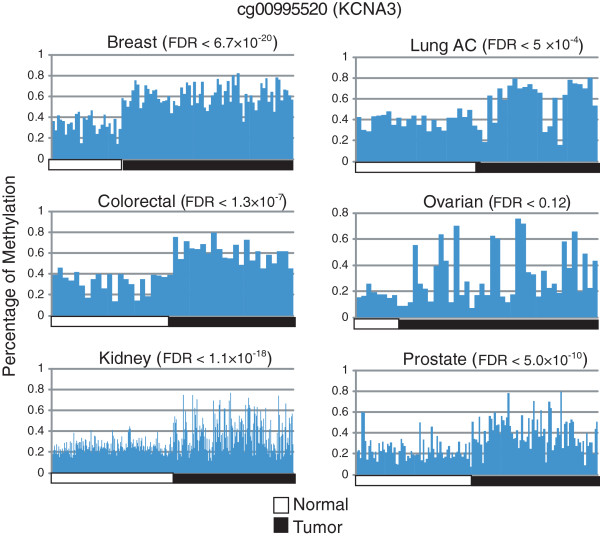
**Hypermethylation in the KCNA3 promoter region across multiple cancer types.** Voltage gated channel activity is highly enriched with hypermethylated genes. The silencing of potassium voltage-gated channel, shaker-related subfamily, member 3 (KCNA3) via promoter methylation has been reported in breast and pancreas tumors. Here, we observe KCNA3 promoter methylation as a prevalent event across multiple cancer types including lung, ovarian, kidney, prostate, and colon. FDR levels were calculated using LIMMA package as described in *methods*.

LRpath analysis also revealed voltage-gated channel activity is significantly altered across multiple cancer types (FDR < 0.003 in 5 tumor types). The concepts involved in voltage-gated potassium channel activity include genes such as *KCNQ1*, *SNAP25*, *KCNA3*, and others, which are known to play a role in cell proliferation. Further downstream analysis reveals *KCNA3* promoter hypermethylation in 8 out of the 10 cancers, identifying it as one of the most prevalent events in tumorigenesis and affecting various tumor types (Figure 3).

**Figure 4:**
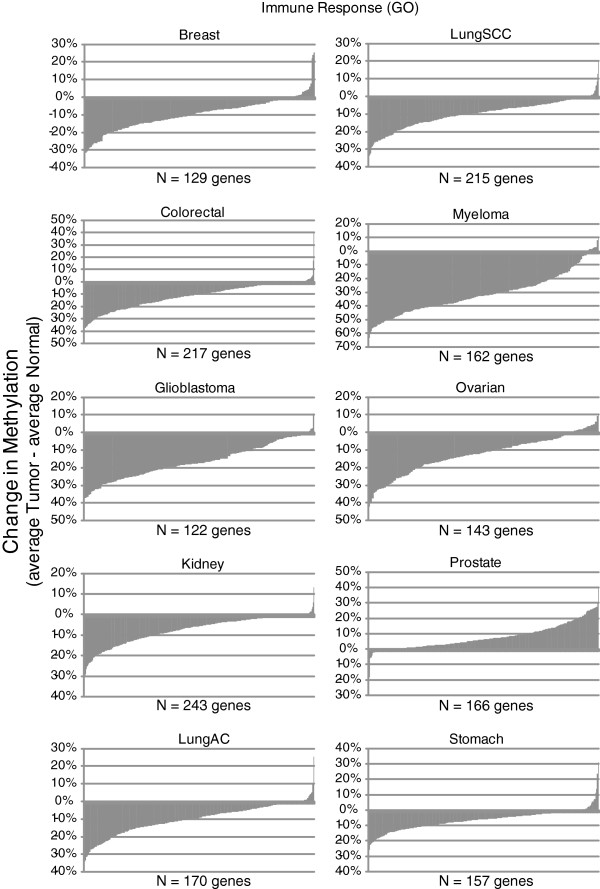
**Difference in methylation in significant genes between normal and tumor samples involved in immune response (GO term).**Waterfall plots of the average methylation change of those genes involved in immune response (average methylation % among tumor samples - average methylation % among normal control samples) is shown below. All except prostate cancer show a significant number of genes undergoing hypomethylation, a possible mechanism for gene activation.

The majority of hypomethylated gene sets identified across multiple cancer studies were immune-related concepts such as chemokine (FDR < 0.002 in 5 tumor types), cytokine (FDR < 0.02 in 5 tumor types), receptor binding activities (FDR < 0.02 in 5 tumor types), responses to stimulus (FDR < 0.04 in 5 tumor types), and inflammation (FDR < 0.005 in 5 tumor types) (Figure 4). The epidermis (FDR < 2.7×10^-9^ in 5 tumor types), intermediate filament (FDR < 0.0003 in 5 tumor types), and keratin concepts (FDR < 6.6×10^-5^ in 5 tumor types) involved in ectoderm development also form a tight cluster of hypomethylated concepts, suggesting DNA methylation-driven cancer cell invasion and tumorigenesis across various types of cancer (Additional file 2: Figure S1B). Finally, genes involved in peptidase activity (FDR < 0.06 in 5 tumor types) had a significant tendency to be hypomethylated across cancer types.

**Figure 5:**
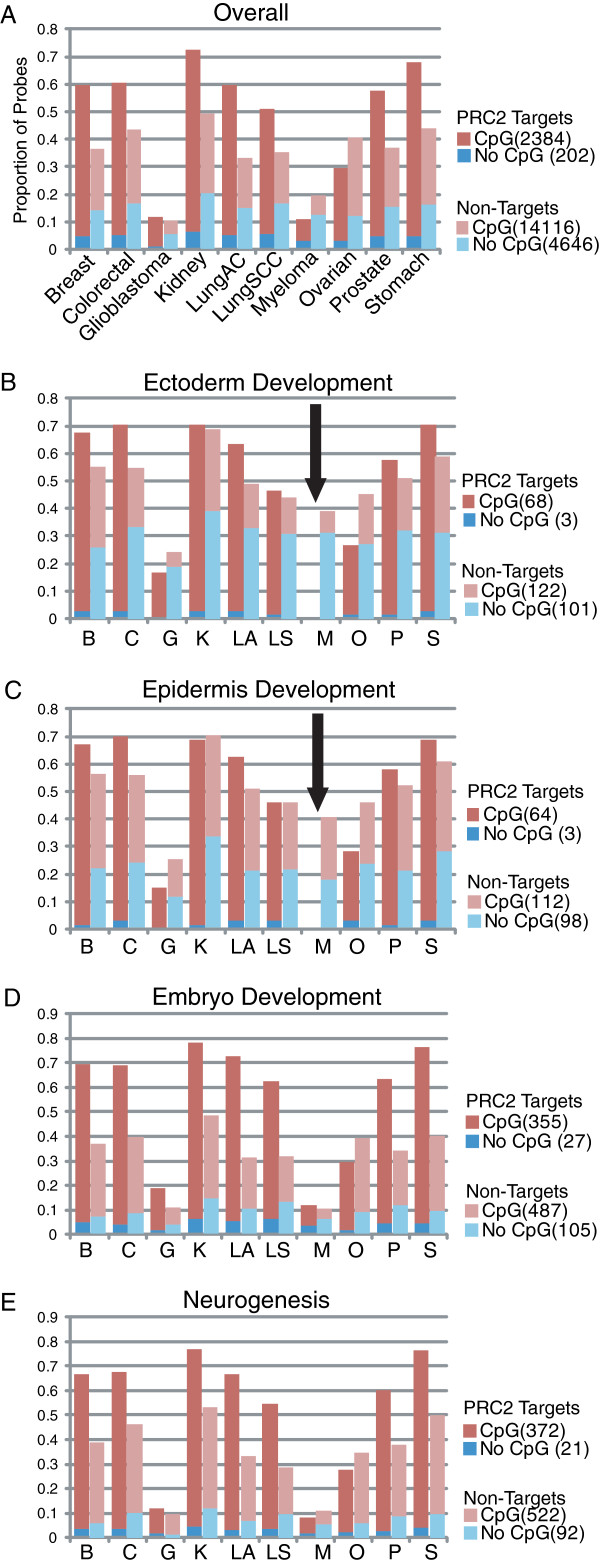
**The percentage of PRC2 target vs. non-target genes harbouring significant (p-value<0.05) differential methylation.** The significant differentially methylated genes from a select few developmental concepts including dermal, embryo, and neural development were subjected to further analysis with respect to PRC2 targets and the presence of CpG islands. As reported, a higher proportion of PRC2 target genes was differentially methylated in multiple tumor types. Interestingly, none of the PRC2 target genes involved in dermal development were differentially methylated in multiple myeloma.

We examined the significant genes from Ectoderm (FDR < 0.0003 in 5 tumor types) and Epidermis development (FDR < 7.7×10^-5^ in 5 tumor types) in each cancer type in the context of the occupancy of PRC2 components SUZ12 and EED, and H3K27me3 [25]. The majority of the genes involved in these pathways that are bound by these PRC2 proteins exhibit differential methylation (Figure 5 and Additional file 2: Figure S4 and 5A-D). The ectoderm and epidermis development pathways were shown to be enriched with hypomethylated genes, which was driven by the non-PRC2 targets; the PRC2 target genes in these pathways were more prone to be hypermethylated (Additional file 2: Figure S5A and 5B). In contrast, the pathways involved in embryo development and neurogenesis were enriched among hypermethylated genes, and both PRC2 and non-PRC2 targets showed a higher proportion of hypermethylated genes, although the trend seemed stronger among the PRC2 targets (Additional file 2: Figure S5C and 5D). Interestingly, while around 40% of non-PRC2 target genes involved in ectoderm and epidermis development were differentially methylated in multiple myeloma (comparable to the other types of cancers), none of the PRC2-target genes are significantly differentially methylated in multiple myeloma. 

Additional concept types available in LRpath include metabolite concepts that combine metabolic enzyme coding genes, DrugBank concepts, and transcription factor targets (see *Methods* for details). In our directional analysis we found several metabolite concepts that were consistently enriched across cancer types. The hypomethylated concepts included several metabolite concepts in androgen and estrogen metabolism, C21-steroid hormone biosynthesis and metabolism, tyrosine metabolism, and xenobiotics metabolism (Additional file 2: Figure S6A). Genes involved in these concepts encode several prominent groups of enzymes including multiple members of the Cytochrome P450 family, steroid biosynthesis enzymes and members of the UDP glucuronosyltransferase family. The hypermethylated metabolite concepts included cyclic AMP (cAMP) and cyclic GMP (cGMP) which include genes encoding several phosphodiesterases and adenylate cyclases (Additional file 2: Figure S6A). In addition, we identified twelve Drug Bank concepts, each of which consists of genes known to interact with a specific drug (Additional file 2: Figure S6B). Several transcription factors were predicted to target genes enriched with hypermethylation across cancer types, including AHR-ARNT, ATF2 (CREBP1), PAX4, E2F2 and NRSF (Additional file 2: Figure S6C).

In addition to clustering pathways and other biological concepts significant across several cancer types, we also performed clustering on biological concepts significant in any one or more cancer types (Figure 2- right side). The two heatmaps in Figure 2 look surprisingly similar, suggesting that the majority of pathways affected by DNA methylation in cancer are common to multiple cancer types.

### Identification of biological concepts enriched or depleted in genes dysregulated via CpG methylation across cancer types (Non-directional LRpath analysis)

**Figure 6:**
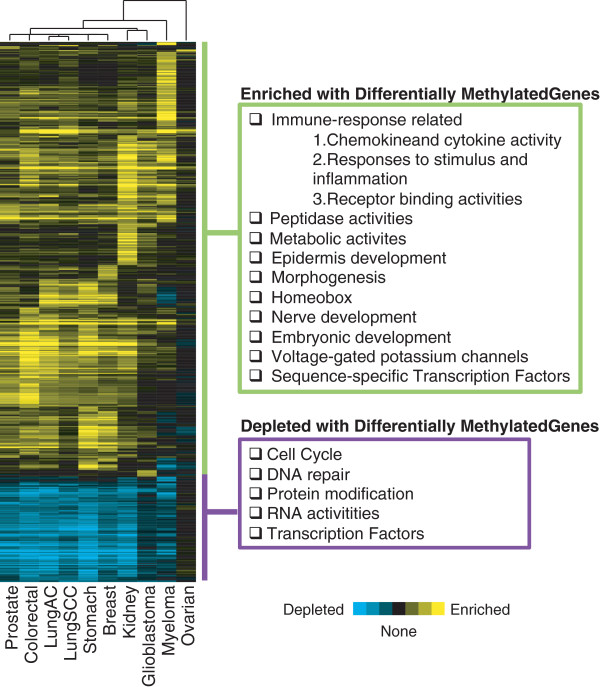
**Hierarchical clustering of significant biological concepts from non-directional LRpath tests.** The data from 10 different cancer studies were subjected to non-directional LRpath analysis and then clustered using criteria indicated in the figure. The clustering of LRpath results suggests that similar pathways across multiple cancers are consistently depleted or enriched by the differentially methylated genes. Concepts such as DNA repair and cell cycle activity, which play a crucial role in cancer development and progression, are depleted in differentially methylated genes (Criteria: At least one study with p-value < 0.00001, N=661 genes).

Similar to the directional analysis results performed on ten tumor versus normal methylation studies, the clustering analysis of GO terms and pathways from *non-directional* LRpath analysis also exhibited tightly clustered enriched and depleted concepts across multiple cancer types (Figure 6). DNA repair (FDR < 0.0005 in 5 tumor types) and cell cycle activity (FDR < 0.016 in 5 tumor types), two of the most commonly affected pathways in cancer development, were depleted in differentially methylated genes. To determine if notable exceptions to this trend exist, we examined the change in methylation of those genes involved in cell cycle. Among the 564 unique genes (1,101 total probes) related to cell cycle pathways on the Illumina BeadChip, 42 genes including several key regulators such as *APC*, *CDKN2A* and *2B*, and *RASSF1* harboured greater than 20% average methylation change in at least 3 tumor types (30 hypermethylated and 12 hypomethylated genes as seen in Additional file 2: Figure S7).

Similarly, among the total of 237 unique genes (450 probes) related to DNA repair on the Illumina BeadChip, only 10 hypermethylated and 13 hypomethylated genes with greater than 15% change in average methylation in at least 3 studies were identified. These included the p53 related gene, p73 (*TP73*) [26] and DNA repair protein O6-methylguanine-DNA methyltransferase (*MGMT*) involved in DNA repair activity. Interestingly, patients with *MGMT* hypomethylation were shown to have worse survival compared to those with *MGMT* promoter methylation (12.2 months vs. 18.2 months) [27].

Although the concepts involved in cell cycle and DNA repair activity were shown to be depleted in differential methylation, indicating fewer genes involved in this concept are affected via DNA methylation change than by chance, certain crucial regulator genes such as *APC*, *CDKN2A*, and *CDKN2B*[21,28,29] were still shown to be differentially methylated to a great extent in multiple tumor types. As seen in Additional file 2: Figure S7, one of the *APC* probes was hypermethylated by more than 10% in 5 out of 10 tumor types, and probes for *CDKN2A* and *CDKN2B* genes were hypermethylated by more than 10% in all 10 types.

### Overlap among differentially methylated genes in enriched biological concepts across cancers

The same significant pathways could be affected by either similar or different sets of methylated genes across various cancer types. The concepts involved in epidermis development, immune response, and neurogenesis were three of the most commonly affected significant concepts (Figure 2 and 4; Additional file 2: Figure S1A and 1B). Based on Fisher’s exact tests for non-random associations between any two studies from the ten data sets (resulting in 44 pairs), mostly the same genes appeared to be driving enrichment. In those concepts involved in epidermis development and immune response, which were both enriched with hypomethylated genes, every pair except those paired with prostate cancer were highly significant (Additional file 1: Table S1). Neurogenesis was enriched among hypermethylated genes, and again we saw a high degree of overlap among the specific genes determining enrichment. While the prostate study seemed to be consistent with other cancer types for neurogenesis, the myeloma and ovarian studies tended not to be significant. In myeloma, very few genes involved in neurogenesis were differentially methylated (N = 5 genes) in comparison with other studies (which ranged from 30 to 231 genes in other types), thus non-correlation observed in myeloma can be explained by the lack of genes involved in neurogenesis.

### Notable cancer-specific results

Although the clustering analysis revealed that most of the significant concepts were shared across multiple types of cancers, several notable cancer type-specific exceptions were observed. First, we identified cancer-specific results from non-directional LRpath results. In glioblastoma, pathways involved in bone morphogenetic protein (BMP) (FDR < 0.0003) were enriched with differentially methylated genes. The importance of BMPs in glioma was previously studied *in vivo* using glioma stem cells treated with BMPs, which effectively delayed tumor growth and reduced tumor invasion [30]. In prostate cancer, extracellular related concepts (such as extracellular region part (FDR < 4×10^-20^), extracellular space (FDR < 7×10^-15^), extracellular matrix (FDR < 5×10^-10^), and proteinaceous extracellular matrix (FDR < 3×10^-9^)) and adhesion related concepts (FDR < 1×10^-8^) were significantly enriched among hypermethylated concepts, compared to others. To identify additional concepts that are highly cancer-type specific, the biological concepts significant with p-value < 0.0001 in just one type of cancer in the directional LRpath analysis were examined (Additional file 2: Figure S8). In myeloma, multiple kinase activities (FDR < 0.0014) were hypermethylated, and muscle/fiber related concepts (FDR < 9×10^-5^ for contractile fiber part) were hypo-methylated. In breast cancer, several processes involving circadian rhythms (FDR < 0.017) were hypermethylated.

## Discussion

Performing an integrative analysis of biological concepts dysregulated via methylation across ten cancer types, we identified concepts affected in multiple cancer types that support biologically important findings. The underlying logistic regression method used by LRpath has been shown to perform favorably [13]. The current application of our LRpath web-based software allowed us to not only identify pathways regulated via hyper- or hypo- DNA methylation for each cancer type, but to also determine biological concepts depleted in DNA methylation changes and to easily integrate and visualize the results. In addition, an important feature of LRpath that distinguishes it from many other programs is the availability of a broad range of concept types such as transcription factor and drug targets, metabolites and literature-derived concepts that are not available in other programs. These concepts are often smaller than commonly used GO terms or pathways and have potential to point to very specific changes in metabolism or a regulatory process.

### Hypomethylated biological concepts

Because the available data are reflective of tumor cellular heterogeneity, aberrant methylation of certain pathways is generally reflective of a heterogeneous cell population that includes the tumor environment. It’s worth noting that such information would be lost if analysing cell lines with 100% cellularity, and may be particularly relevant to the identification of clinically relevant biomarkers of risk and prognosis. For example, inflammation, which was hypomethylated across cancers, is a marker of senescence which plays a major role in the tumor microenvironment. As a key element in cancer progression, senescence allows an influx in inflammatory elements into tumor cells causing tumorigenesis at multiple levels: DNA damage, cell survival, angiogenesis and promotion of growth [31]. Chemokine and cytokine activity further promote inflammation. Peptidase activity, which was also hypomethylated across cancers, is required for the tumor cells to break through the extracellular matrix and basement membrane barriers to becoming invasive, and thus its predicted up-regulation via hypomethylation would promote metastasis [32]. Other hypomethylated concepts, epidermal and keratinocyte development and differentiation, have been linked to worse survival prognosis and increased local invasiveness [33].

As shown in the results, the majority of hypomethylated concepts are related to immune response, and promoter DNA hypomethylation often results in gene activation. This inflammatory activation via DNA hypomethylation could be due to an influx of lymphocytes into the tumor microenvironment or due to a difference in the DNA methylation in the tumor cells themselves. While it is beyond the scope of this manuscript to conclude to what extent each of the above possibilities contributed, regardless of the origin of the inflammatory response, we speculate the change in DNA methylation is a common mechanism to elevate immune responses across multiple cancers.

Identification of metabolite concepts that include members of the Cytochrome P450 (CYP) and UDP glucuronosyltransferase (UDPG) families suggests that promoter hypomethylation may be involved in regulation of their transcript levels. CYP proteins have been shown to be expressed across multiple tumor types [34]. CYP enzymes mediate the metabolic activation of numerous precarcinogens, and they can promote or suppress tumor development via hormonal control in cancers that are sensitive to hormone concentration (e.g. breast cancer). UDP glucuronosyltransferases catalyse the glucuronidation of many lipophilic endogenous and exogenous substrates such as bilirubin, estrogens, and xenobiotics. These enzymes, along with ABC transporters, are involved in multiple drug resistance, and their expression is also often altered in cancers.

### Hypermethylated biological concepts

Among hypermethylated gene groups, which we predict would be down-regulated in tumors, were nervous system and embryonic development genes. We observed a high degree of overlap between these concepts and Polycomb Repressive Complex 2 (PRC2) target genes. The group of genes regulating early development, normally regulated by PRC2, often becomes methylated in cancer [35]. Even in ectoderm and epidermis developmental pathways that are enriched with hypomethylated genes, the PRC2 targets tended to be hypermethylated (Additional file 2: Figure S5A and 5B). Interestingly, the cancer type that displayed the lowest number of hypermethylated PRC2 targets was multiple myeloma, the only non-solid tumor analysed, despite having the second highest number of differential methylation sites (below colorectal cancer) (Table 1 and Figure 5). Unlike the other nine cancers examined in this study, multiple myeloma is a blood cell cancer, and the absence of differential methylation among PRC2 target genes involved in early development and morphogenesis pathways may be due to the different nature of cancer development and invasion in blood cancer. Downstream analysis at the gene level also identifies multiple myeloma as the most divergent cancer from the rest. Based on Fisher’s Exact tests for non-random associations between any two studies from the ten data sets (resulting in 44 pairs), there appear to be mostly the same genes driving enrichment in neurogenesis, (every pair except those involved in myeloma data are highly significant) (Additional file 1: Table S1).

Voltage-gated potassium channels, hypermethylated in tumors, play various roles in cancer progression, such as its initial role during the onset of the disease, as well as cell proliferation, apoptosis, migration, and invasion during metastasis [36]. The gene inactivation via promoter DNA methylation events in voltage gated gene Kv1.3 (*KCNA3*) has been previously reported in breast and pancreas adenocarcinomas [37,38]. Our analysis validated *KCNA3* as hypermethylated in breast cancer, plus identified it as hypermethylated in an additional 7 tumor types. Another example is human ether-a-go-go-related gene 1 (*hERG1*), which we found significantly differentially methylated in lung adenocarcinoma, myeloma and stomach cancers. *hERG1* is often dysregulated in cancer and physically interacts with integrin to modulate adhesion dependent intracellular signalling cascades, including cell adhesion, invasion, and proliferation [39,40].

When the biological concepts enriched in just 1 type of cancer (p-value < 0.0001) are examined, the enrichment of genes involved in circadian rhythm was identified in breast cancer. The disruption of normal circadian rhythm might benefit the survival of cancer cells, and the circadian rhythm disruption has been proposed as a risk factor for breast cancer [41]. Promoter hypermethylation concomitant with a decrease in expression was identified for the circadian genes *PER1* and *PER2* in breast cancer [42]. Based on our LRpath results, we identified additional circadian genes, *DRD1* (FDR < 9.9×10^-7^), *CASP1* (FDR < 0.002), *PTGDS* (FDR < 4.8×10^-23^), and *PGLYRP1* (FDR < 8.5×10^-7^) as hypermethylated in breast tumor samples (significance levels based on probe-level LIMMA analysis, see *Methods*); these genes play a role in the regulation and disruption of circadian rhythm (Additional file 2: Figure S9).

Transcription factors, as a group represented by the sequence-specific DNA binding and homeobox concepts, also tended to be hypermethylated. There are a number of transcription factors commonly hypermethylated in our analysis including the *HOX* gene family, *FOX* gene family, *PAX* gene family, the tumor suppressor *WT1*, and others. The vast majority of the genes involved in transcription factor activity were PRC2 targets (Additional file 2: Figure S3), which confirms the high degree of overlap between PRC2 target genes and those that are methylated in cancers.

Among the hypermethylated metabolite-centered concepts, cyclic AMP (cAMP) is of interest, because it is a key second messenger involved in numerous cellular events. In cancers, cAMP analogues are known to decrease the rate of proliferation of cells and induce apoptosis [43].

### Biological concepts depleted in genes with aberrant methylation

From non-directional LRpath tests and clustering, we determined that DNA repair and cell cycle had fewer differentially methylated genes than expected by chance. We hypothesize that genes involved in DNA repair and cell cycle tend to be dysregulated by alternative mechanisms such as genomic aberrations, somatic mutations, or histone modifications. Alternatively, dysregulation of these pathways could be driven by single key genes with large effects, which would not be revealed in a pathway level analysis. To test the presence of differential methylation in a select set of key regulator genes, we examined individual methylation levels of all genes involved in either DNA repair or cell cycle. While the majority of the genes did lack differential methylation, we found that certain crucial key regulator genes of cell cycle such as *APC*, *CDKN2A* and *CDKN2B*[28,29,44] are indeed hypermethylated across most tumor types and had an average difference in methylation of at least 20% for three or more cancers (Additional file 2: Figure S7). Likewise, *MGMT* and *TP73* exhibit hypomethylation in multiple tumor types. Thus, although few genes in cell cycle and DNA repair are affected by differential DNA methylation, many that are affected are known key driver genes in cancer.

### PRC2 target genes involved in early development

Concepts involved in early development (such as ectoderm, epidermis, and embryonic development, and neurogenesis) were commonly identified as differentially methylated in our LRpath analysis. Interestingly, some tended to be hypomethylated (ectoderm and epidermis) while others were hypermethylated (embryonic and neurogenesis). Since many of the genes involved in early development are reported to be regulated by PRC2 and are the targets of methylation, we examined these genes under the context of PRC2 targets and the presence of CpG islands (Additional file 2: Figure S4). Whether they are PRC2 targets or not, the percentage of significantly altered genes involved in the above four developmental pathways is slightly higher than what is expected by chance (Figure 5). As expected, PRC2 targets contain a higher percentage of differentially methylated genes than non-PRC2 targets with few exceptions (glioblastoma and myeloma in ectoderm development; glioblastoma, myeloma and ovarian in epidermis development; ovarian in embryo development; and myeloma and ovarian in neurogenesis). While the non-PRC2 target genes located outside of CpG islands involved in ectoderm and epidermis development (hypomethylated concepts), show an increased proportion of methylation change, this is not seen in non-PRC2 target genes located outside of CpG islands involved in embryo development and neurogenesis (hypermethylated concepts).

Interestingly, while around 40% of non-PRC2 target genes involved in ectoderm and epidermis development were differentially methylated in multiple myeloma (comparable to the other types of cancers), none of the PRC2-target genes are significantly differentially methylated (Black arrows from Figure 5). We speculate the absence of differential methylation among PRC2 target genes involved in early development and morphogenesis pathways may be due to the different nature of cancer development and invasion in non-solid tumors.

## Conclusions

Besides its role in suppressing repeat elements in the genome, DNA methylation has evolved to regulate certain biological phenomena that need to change within an individual’s lifetime (e.g., development and differentiation, response to environment), yet still retain a certain level of stability [45,46]. Therefore, one could predict that dysregulation of DNA methylation in cancers would tend to occur in the types of biological processes that require this level of control, for example immune system and cell differentiation [46]. Several specific pathways in these broad categories, also known to be involved in cancer, were identified in this study. On the other hand, other pathways constitutively required by most cells, would not be predicted to be regulated via DNA methylation. Several such pathways, for example DNA repair and cell cycle, were either depleted or saw no significance in the number of genes with differential methylation even though some such pathways are known to be important in cancer development and progression. We hypothesize that these pathways tend to be dysregulated by genetic alterations and/or alternate epigenetic mechanisms, or by key regulator genes. Our analyses may also reflect methylation events that are involved solely in cancer progression as opposed to initiation. A similar analysis of early lesions or precancerous tissue may result in different gene sets, since the methylation status of genes is labile. Based on the results of our integrative analysis, we conclude that regardless of tumor type, similar pathways are affected by aberrant CpG methylation during carcinogenesis. Although many of the observed methylation changes may not result in a change in gene expression, such methylation changes, when consistent, may still serve as biomarkers of prognosis. Further studies will shed light on consistent differences between solid and non-solid tumors in terms of DNA methylation.

Although we found that many of the same genes exhibited aberrant promoter DNA methylation across cancers, which of these specific changes drive cancer development and progression may differ to a greater extent among cancer types. Such differences are likely due to tissue-specific expression and functions. Thus, further studies are required to elucidate which players tend to be the drivers of each cancer type. A second limitation of this study is the limitation of assessed sites to those present on the Illumina HumanMethylation27 BeadChip, which are focused mainly in or near CpG islands and in gene promoter regions. Thus, if a pathway tends to be regulated via differential methylation mainly outside of CpG islands, it may be missed in the present study. Comprehensive analysis of rapidly emerging studies performed using reduced representation bisulfite sequencing (RRBS) and whole genome bisulfite sequencing (WGBS) will clarify this issue.

## Methods

### Biological concept database

LRpath uses an internal annotation database that contains a wide variety of gene sets (concepts) representing several types of biological knowledge, and based on the database used by ConceptGen ( http://conceptgen.ncibi.org) [1]. Based on the original data source for each group of concepts, the concepts were grouped into the following categories: functional annotations, literature derived concepts, target sets, interactions, metabolite-centered concepts and chromosomal location (Cytoband) (Additional file 1: Table S1). Data were downloaded from respective sources. To build the transcription factor targets concepts, KnownGene, KnownToLocusLink, and TfbsConsSites tables were obtained from UCSC Genome browser (Mar. 2006, NCBI36). For each known gene, the Entrez Gene ID (formerly known as Locus Link ID) is assigned using the KnownToLocusLink table, and the list of transcription factors that bind to a gene promoter region (±2,000 bp of TSSs) was generated using minimal overlap.

For miRNA concepts, the TargetScanS table containing 54,199 conserved miRNA target sites in human Refseq genes predicted by TargetScanHuman5.1 was obtained from the UCSC genome browser, as well as the target sites predicted by miRanda using the newly improvised mirSVR algorithm from microRNA.org. The latter source has predicted over 16 million miRNA target sites in 34,911 distinct 3^′^UTR in human, and for downstream analysis, 3,155,472 non-conserved and 1,047,672 conserved miRNA sites (totalling approximately 4 million sites) with good mirSVR scores to be considered further. To provide high-quality miRNA targets, only the target sites predicted by both targetScan and miRanda algorithms within 3^′^UTR regions of the human genome were included in the LRpath database, covering 36,015 sites with 153 different miRNAs. The other concepts were created as described previously for ConceptGen software (Sartor et al., 2010) (Table 2).

**Table 2:** Biological concepts represented in the LRpath database

**Biological knowledge type**	**Concept type**	**Number of concepts**
Functional Annotations	Biocarta pathways	103
	EHMN Metabolic pathways	59
	GO Biological Process	4566
	Go Cellular Component	634
	Go Molecular Function	1264
	KEGG Pathway	222
	Panther Pathway	98
	PFAM	767
Literature Derived	MeSH	6001
	OMIM	142
Targets	Drug Bank	293
	miRBase	149
	Transcription Factors	241
Interaction	Protein Interaction (MiMI)	8999
Other	Metabolite	959
	Cytoband	1151

### Creation of the LRpath application

The LRpath application consists of the web-based user interface, the request handler (Executor), the Rserv (R server) host and the database server. The web interface allows the user to select and upload the input file, select one or more databases to search against, and set the analysis parameters. The application also provides access to several advanced options including setting the maximum and minimum number of genes in concepts, changing the low and high values for calculating odds ratios, and the significance cut-off for reporting the driving genes. Once the analysis has been completed the application will display the output in a table format. In addition to viewing the output as a web page, users can download the analysis results as tab-delimited text or as an Excel file, which provides an opportunity to sort the results and import them into other programs (e.g. the Cytoscape plug-in visualization software Metscape, http://Metscape.ncibi.org).

Since certain LRpath searches can take several minutes to run, the requests are queued and ran as compute resources become available. Approximate run-times for each database are provided on the web site. Currently the system can handle up to five requests simultaneously. Queued requests are served on a “first come, first served” basis, with current jobs marked as “running”. A monitor URL is assigned to each job, which allows users to check the status of their jobs. The user has an option to provide an e-mail address for notification when the job is running and a link to results. This option is particularly useful if multiple large databases are selected (e.g. GO and MeSH).

### Cross-experiment visualization via clustering in LRpath

LRpath results from multiple experiments may be integrated in order to interactively view and explore the enrichment profiles across experiments. It provides a user-friendly method for filtering, merging, and clustering LRpath results using several options. The input for this part of the application is the set of URLs from previous LRpath analyses to be clustered. The user has the ability to choose the values to be used to cluster, the type of distance matrix method, the type of linkage method for hierarchical clustering, and which biological concepts to include. The output is a set of files to input directly into the widely-used and freely-available TreeView software [47,48]. Here, users can view the hierarchical clustering with each row corresponding to a concept, and each column corresponding to an experiment.

### Reanalysis of publicly available CpG methylation data in cancers

For this study, we selected ten tumor versus normal CpG methylation studies profiled on the Illumina HumanMethylation27 BeadChip, four studies from Gene Expression Obmibus (GEO) and six studies from The Cancer Genome Atlas (TCGA) database based on available sample size (N > 40) and the availability of normal adjacent methylation profiling status (at least three normal samples). To represent a wide spectrum of cancers, all studies, with the exception of lung cancer, which is classified into adenocarcinoma and squamous cell carcinoma, were from unique sites: breast, colon [49], brain [50], myeloma [51], kidney, ovarian [52], prostate [53], and stomach. From 27,543 CpG sites, those sites with missing beta score in any one study were filtered out, and 23,050 sites remained for further downstream analysis. Our analyses included 6 paired and 4 non-paired studies, and using LIMMA package in R software, the differential methylation between tumor and adjacent normal samples was examined using beta scores according to experimental design (paired or non-paired). Resulting p-values were adjusted for multiple-comparison using the false discovery rate (FDR) method.

### LRpath enrichment analyses with cancer versus normal datasets

The data representing 23,050 sites generated from the R statistical analysis was reformatted to contain Entrez gene IDs, p-values, and fold-changes in tab-delimited text file format. Fifteen concept types were selected (Biocarta pathway, EHMN metabolic pathway, GO biological process, cellular component, and molecular function concepts, KEGG pathway, Panther pathway, pFAM, MeSH, Drug Bank, miRBase, transcription factors, MiMI, metabolite, and cytoband) for enrichment analysis in LRpath. For each study, the test was performed using both directional and non-directional options with default settings. The link to the final results of each test was received automatically using the email notification functionality.

### LRpath clustering analyses with cancer versus normal datasets

The outputs from the *directional* and *non-directional* tests were subjected to clustering analysis in two separate runs ( http://lrpath.ncibi.org). In *directional* clustering analysis, the links of ten individual studies were used to fill out the web-based analysis form using negative log_10_ p-values, with uncentered Pearson correlation distance matrix and the centroid clustering method. From a total of 8,199 concepts involved in pathways, only 171 concepts remained after filtering using p-value <0.0001 in at least half of the studies criteria, and 139 concepts remained using p-value <1e-11 in at least one study. The first filtering criteria were designed to identify concepts present across multiple tumor types, while the second criteria were for concepts specific to a tumor type. In *non-directional* clustering analysis, a total of 661 concepts involved in pathways remained after filtering to those concepts with p-value < 0.00001. In addition, the significant concepts (at least 3 studies with p-value < 0.001) from directional testing involved in Metabolite, Drug Bank, and Transcription Factors concept types were subjected to clustering analysis using uncentered correlation with centroid linkage. The output files are provided in three formats (atr, cdt, and gtr), and they were visualized using Java Treeview software.

## Abbreviations

(TCGA): The Cancer Genome Atlas; (GO): Gene Ontology; (KEGG): Kyoto Encyclopedia of Genes and Genomes; (MiMI): Michigan Molecular Interactions; (MeSH): Medical Subject Headings; (EHMN): Edinburgh Human Metabolic Network; (UCSC): University of California, Santa Cruz; (LINE): Long INterspersed Elements.

## Competing interests

The authors declare that they have no competing interests.

## Authors’ contributions

MAS conceived of the study and led the software development and analyses. AK co-led the software development and contributed to the concepts data base. JK performed the majority of the analyses. VM, TW, and MP helped to develop the LRpath software. JK, AK, MAS, DD, LR, and TW all contributed to writing the manuscript. All authors read and approved the final manuscript.

## Supplementary Material

Additional file 1 **Table S1.** Significance of overlap in the specific differentially methylated genes in significant GO terms between pairs of studies using Fisher’s exact test (p-value<0.05 is indicated with red text)GO term - Immune Response GO term - Epidermis Development GO term – Neurogenesis. Click here for file

Additional file 2 **Figure S1.** Waterfall plots showing the methylation change in significant genes between normal and tumor samples involved in neurogenesis and epidermis development (GO terms). Positive values indicate hypermethylation in cancer, while negative values indicate hypomethylation in cancer. A. Neurogenesis. B. Epidermis Development. **Figure S2.** Change in average percent methylation of HOX gene family, PAX gene family, and WT1 involved in Transcription Factor Activity. **Figure S3.** Unsupervised clustering of probes involved in Sequence-specific Transcription Factor Activity. **Figure S4.** The status of PRC2 targets and CpG islands for those probes involved in the specified GO terms. **Figure S5.** The proportion of differentially methylated genes among the PRC2 targets and non-PRC2 targets (those probes with the p-value<0.05 and the minimum difference between the average methylation percentage of tumor vs. normal greater than 5% are graphed) A. Ectoderm Development. B. Epidermis Development. C. Embryo Development. D. Neurogenesis. **Figure S6.** Clustering of metabolite, drug target, and transcription factor concepts. Hypomethylated concepts are shown in red and hypermethylated concepts are shown in green. A. Metabolite concepts. B. Drug concepts. C. Transcription Factor concepts.** Figure S7.** Change in average percent methylation of the probes for TP73, CDKN1A, 1B, 1C, 2A and 2B, 2C, 2D, and APC. **Figure S8.** Cancer-specific enriched concepts in LRpath directional analysis. Biological concepts enriched with a significant p-value < 1e-4 in one tumor type are listed in the table below. In myeloma, kinase activities are enriched among hypermethylated genes, and muscle-related processes and components are enriched among hypomethylated genes. In breast cancer, several circadian processes are shown up to be enriched among hypomethylated genes. **Figure S9.** Bar graphs showing the methylation change in genes involved in circadian rhythm process in breast cancer. In tumor samples, the increase in the level of methylation in *DRD1, PTGDS, CASP1*, and *PGLYRP1* genes are observed. Click here for file
