# Supplementary material for: LRpath analysis reveals common pathways dysregulated via DNA methylation across cancer types
Source: BMC Genomics. 2012 Oct 4;13:526. doi: 10.1186/1471-2164-13-526 (PMC3505188; doi:10.1186/1471-2164-13-526)
Supplement: Additional file 1 — Table S1. Significance of overlap in the specific differentially methylated genes in significant GO terms between pairs of studies using Fisher’s exact test (p-value<0.05 is indicated with red text)GO term - Immune Response GO term - Epidermis Development GO term – Neurogenesis. [file 1471-2164-13-526-S1.doc]

Additional file2: Table S1: Significance of overlap in the specific differentially methylated genes in significant GO terms between pairs of studies using Fisher’s exact test (p-value<0.05 is indicated with red text)

A. GO term - Immune Response

B. GO term - Epidermis Development

C. GO term – Neurogenesis
