# Supplementary material for: LRpath analysis reveals common pathways dysregulated via DNA methylation across cancer types
Source: BMC Genomics. 2012 Oct 4;13:526. doi: 10.1186/1471-2164-13-526 (PMC3505188; doi:10.1186/1471-2164-13-526)

Supplementary Figure 1: Waterfall plots showing the methylation change in significant genes between normal and tumor samples involved in neurogenesis and epidermis development (GO terms). Positive values indicate hypermethylation in cancer, while negative values indicate hypomethylation in cancer.

A. Neurogenesis

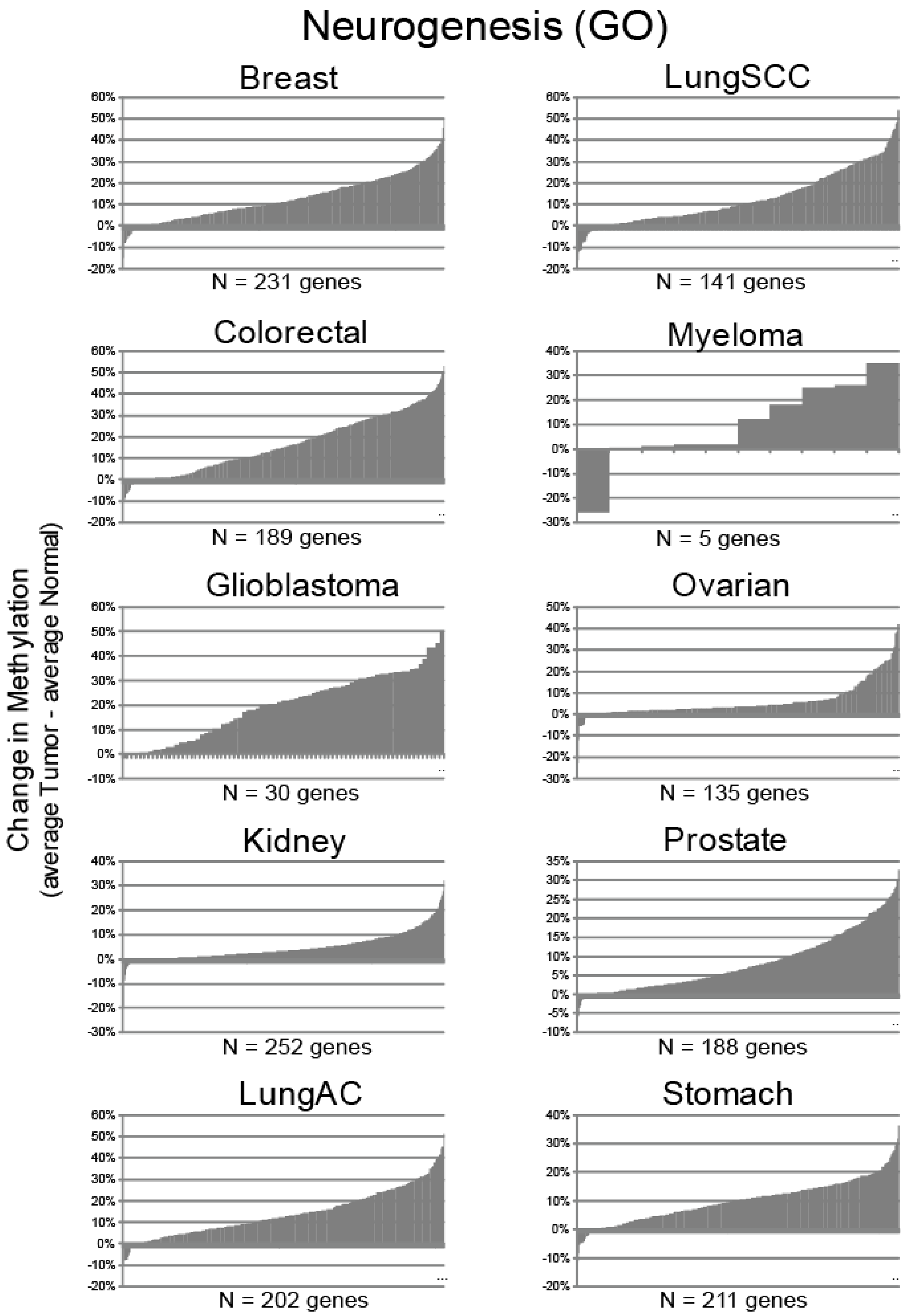

B. Epidermis Development

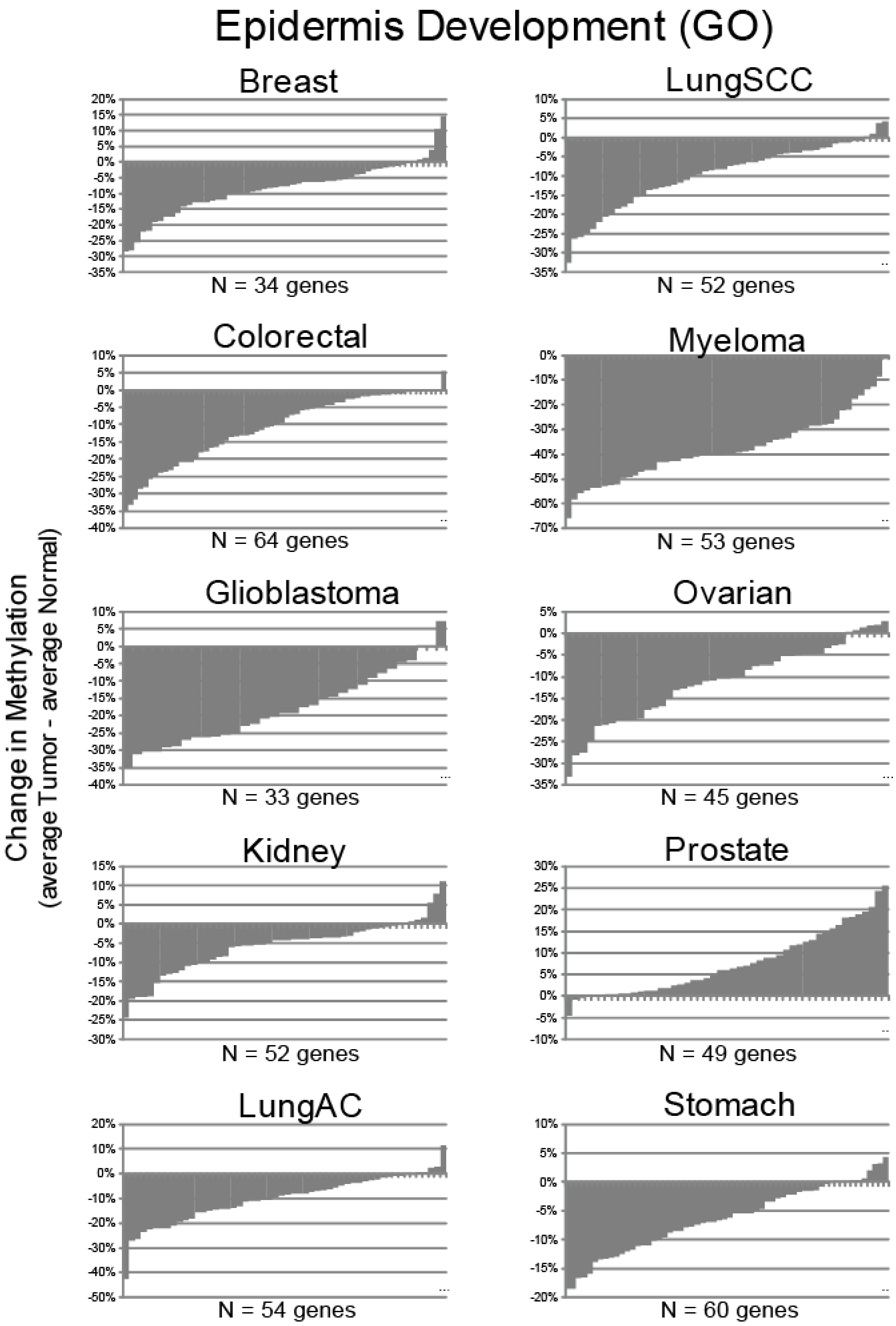

Supplementary Figure 2: Change in average percent methylation of HOX gene family, PAX gene family, and WT1 involved in Transcription Factor Activity

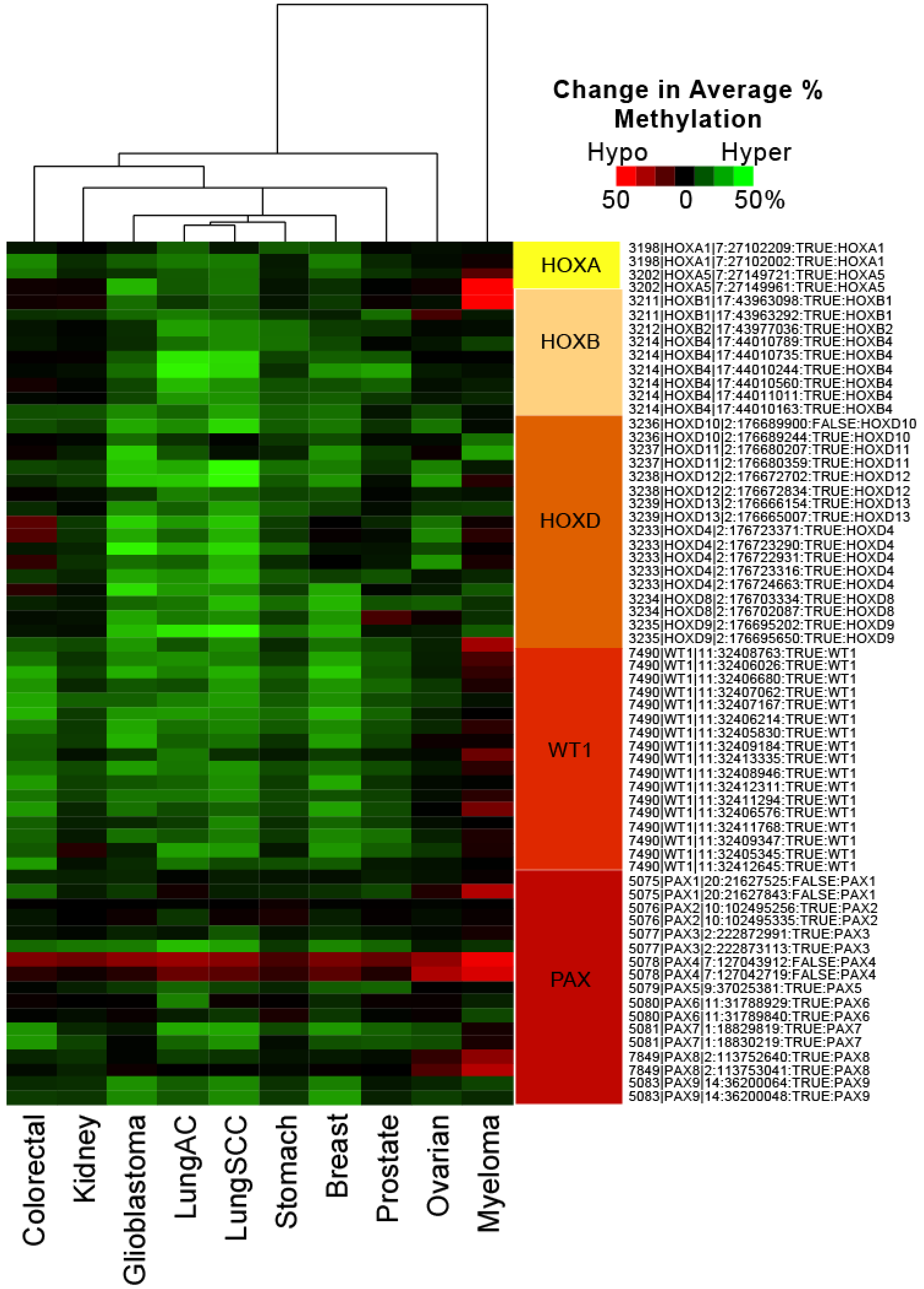

Supplementary Figure 3: Unsupervised clustering of probes involved in Sequence-specific Transcription Factor Activity

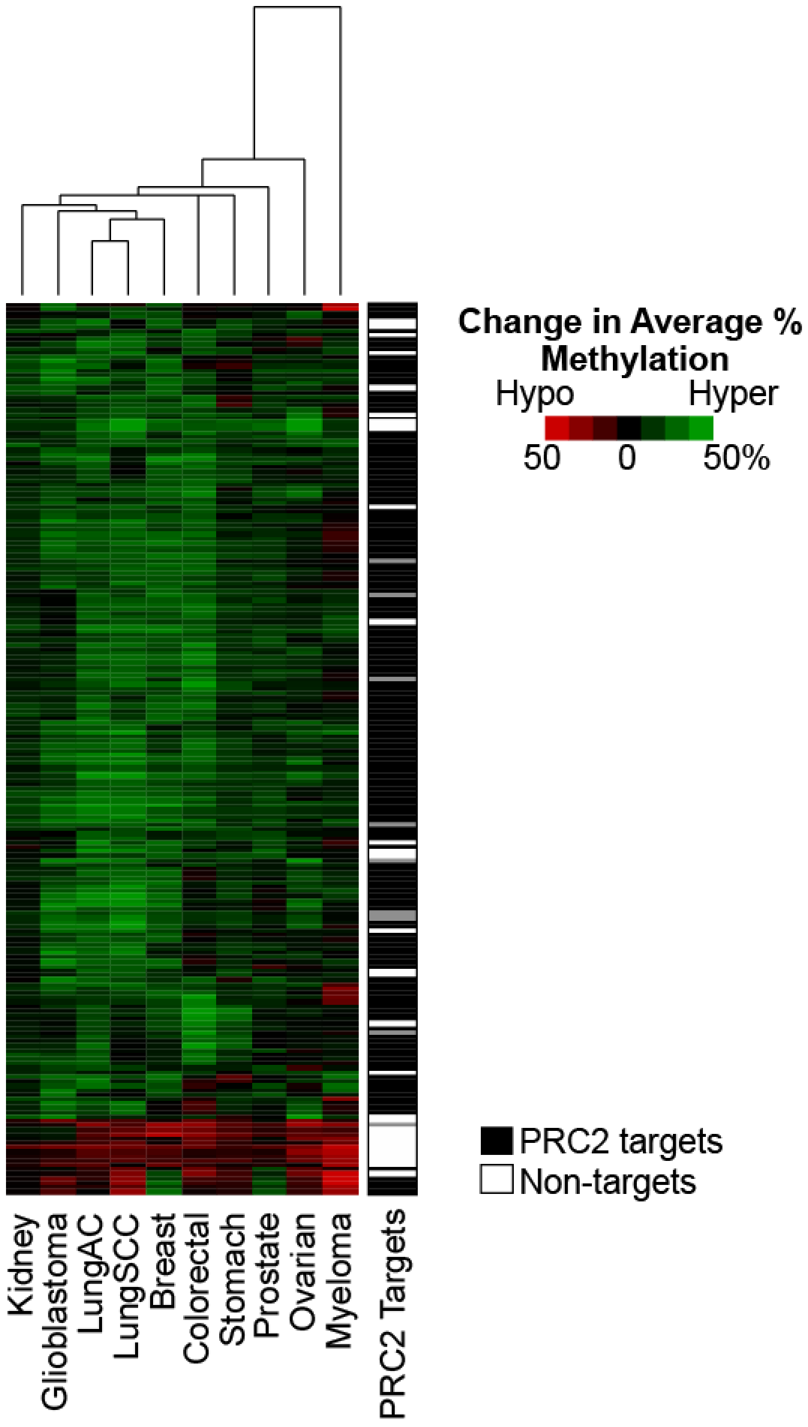

Supplementary Figure 4: The status of PRC2 targets and CpG islands for those probes involved in the specified GO terms

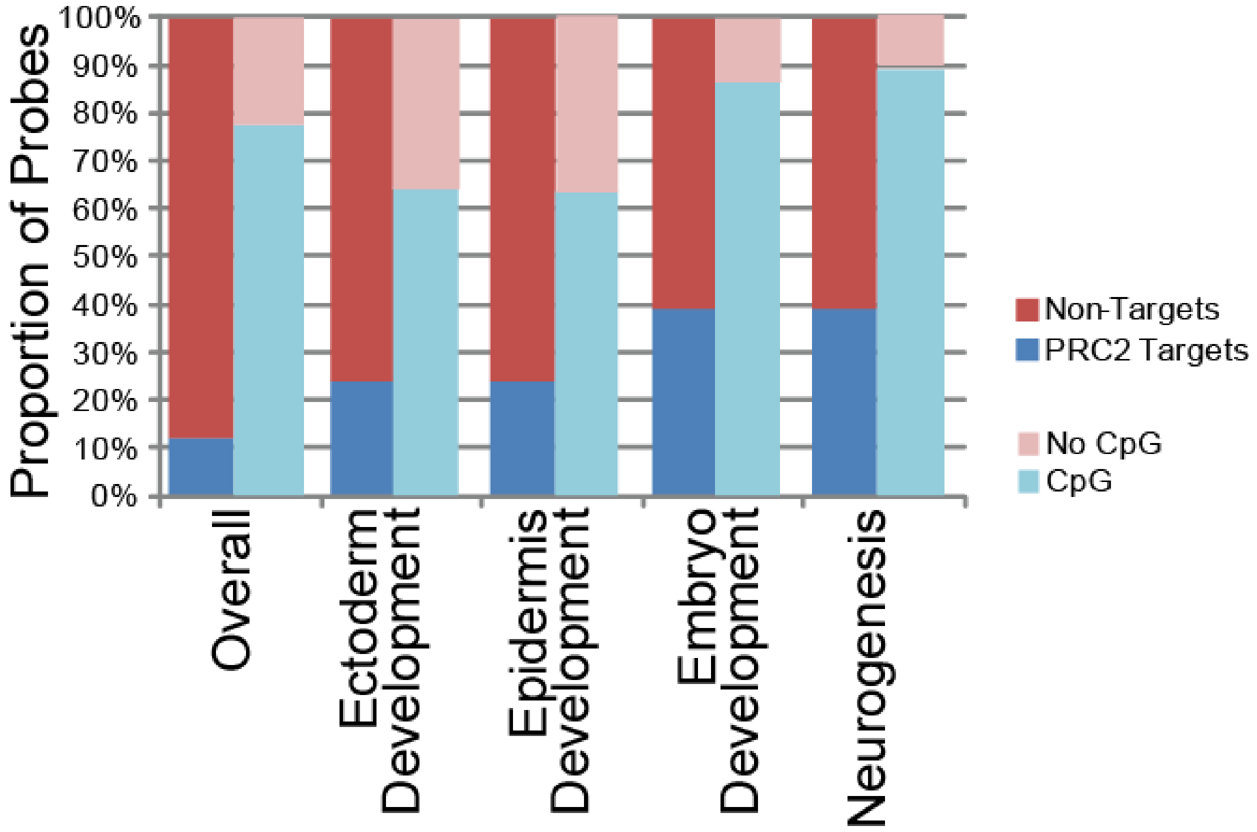

Supplementary Figure 5: The proportion of differentially methylated genes among the PRC2 targets and non-PRC2 targets (those probes with the p-value<0.05 and the minimum difference between the average methylation percentage of tumor vs. normal greater than 5% are graphed)

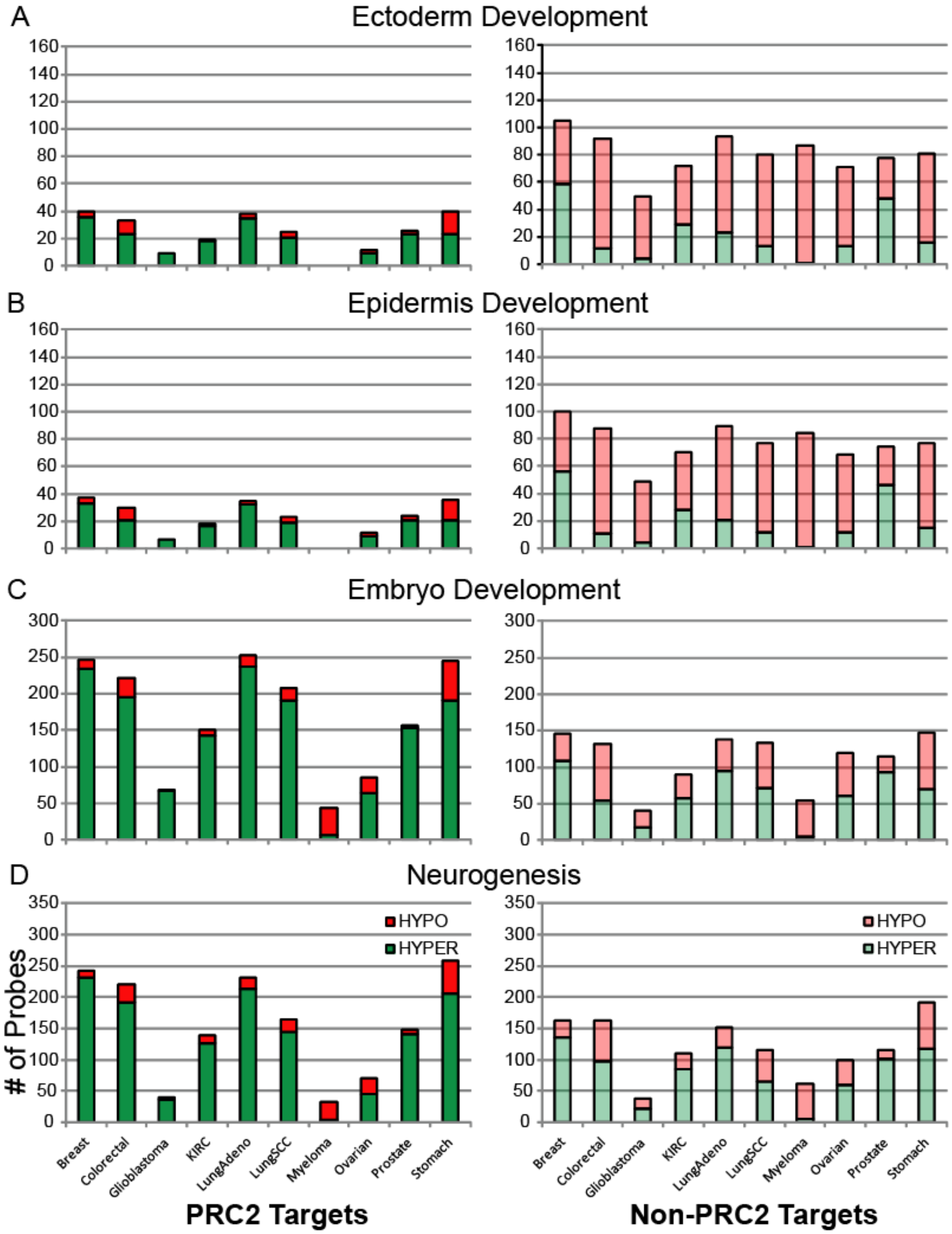

Supplementary Figure 6: Clustering of metabolite, drug target, and transcription factor concepts. Hypomethylated concepts are shown in red and hypermethylated concepts are shown in green. A. Metabolite concepts. B. Drug concepts. C. Transcription Factor concepts

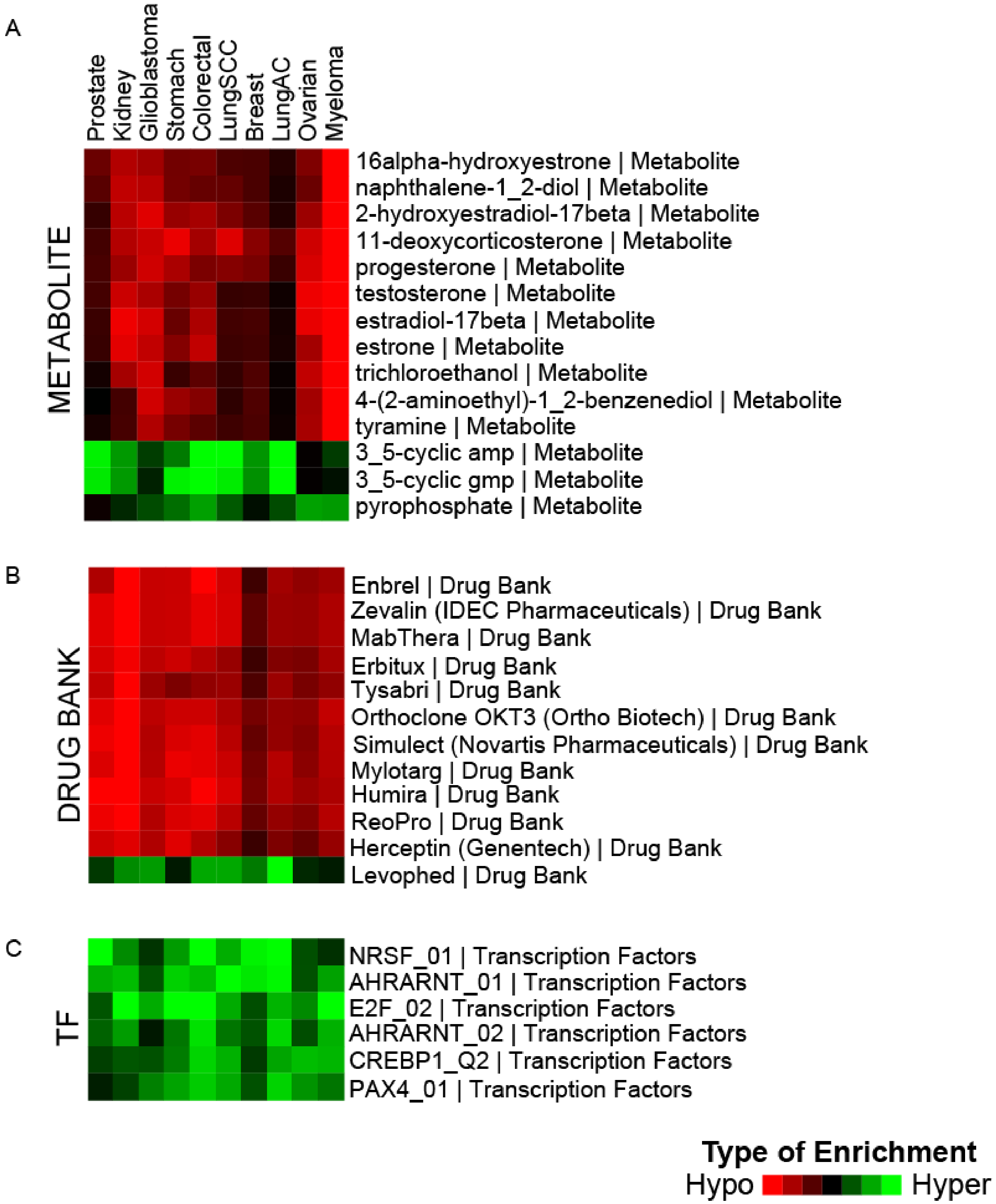

Supplementary Figure 7: Change in average percent methylation of the probes for TP73, CDKN1A, 1B, 1C, 2A and 2B, 2C, 2D, and APC.

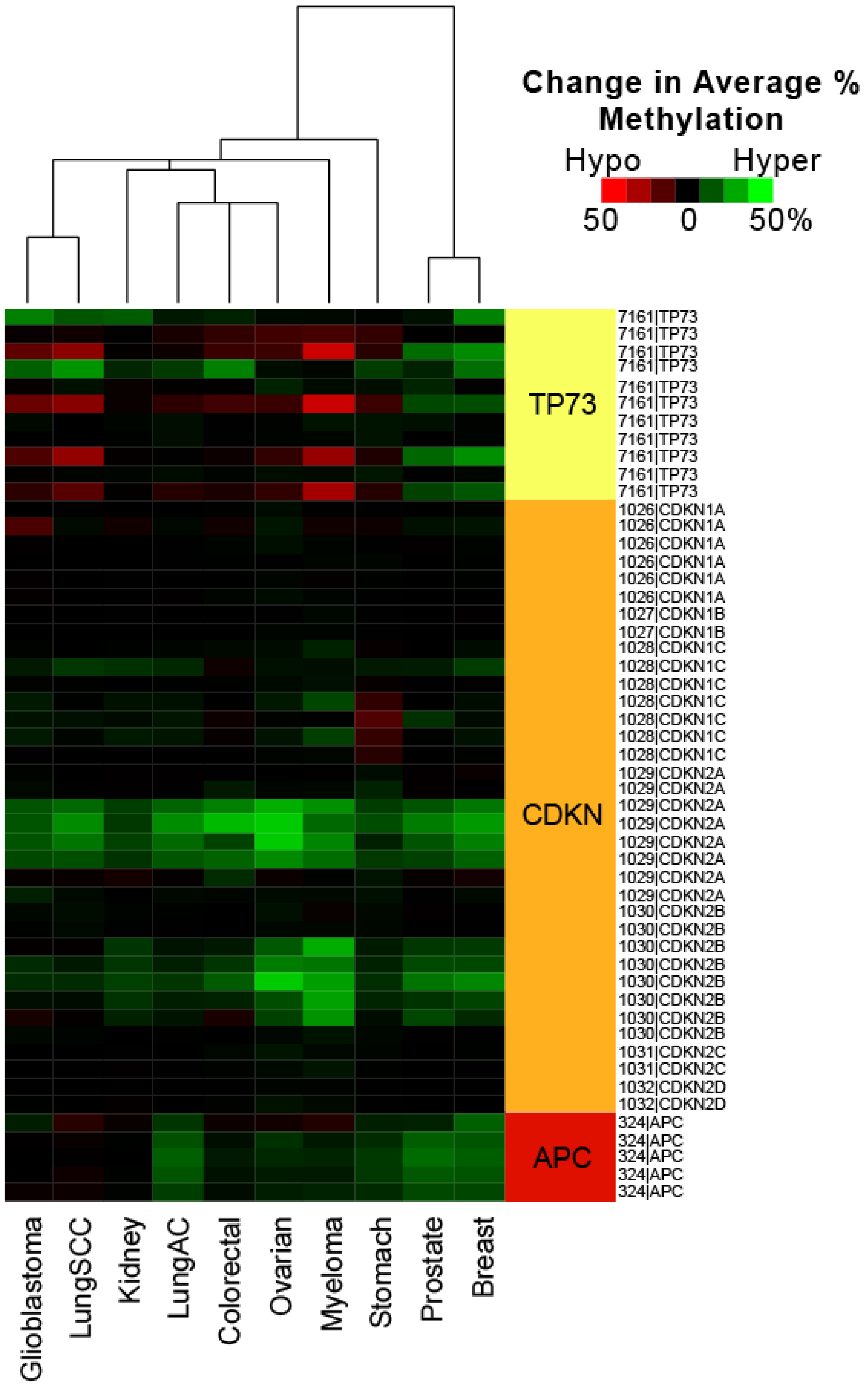

Supplementary Figure 8: Cancer-specific enriched concepts in LRpath directional analysis

Biological concepts enriched with a significant p-value < 1e-4 in one tumor type are listed in the table below. In myeloma, kinase activities are enriched among hypermethylated genes, and muscle-related processes and components are enriched among hypomethylated genes. In breast cancer, several circadian processes are shown up to be enriched among hypomethylated genes.

NAME		Colorectal	Glioblastoma	Kidney	LungAC	LungSCC	Myeloma	Ovarian	Prostate	Stomach	Breast	
kinase activity	GO Molecular Function											Hypermethylated
phosphotransferase activity, alcohol group as acceptor	GO Molecular Function											
transcription cofactor activity	GO Molecular Function											
transferase activity, transferring phosphorus-containing groups	GO Molecular Function											
Protein kinase domain	pFAM											
regulation of cell cycle	GO Biological Process											
circadian sleep/wake cycle process	GO Biological Process											
circadian sleep/wake cycle, sleep	GO Biological Process											
regulation of circadian sleep/wake cycle	GO Biological Process											
regulation of circadian sleep/wake cycle, sleep	GO Biological Process											
regulation of growth	GO Biological Process											
regulation of heart growth	GO Biological Process											
Kazal-type serine protease inhibitor domain	pFAM											
metal ion transmembrane transporter activity	GO Molecular Function											Hypomethylated
cellular amino acid derivative biosynthetic process	GO Biological Process											
regulation of striated muscle contraction	GO Biological Process											
cation transport	GO Biological Process											
contractile fiber	GO Cellular Component											
contractile fiber part	GO Cellular Component											
myofibril	GO Cellular Component											
Pancreatic secretion	KEGG Pathway											

Supplementary Figure 9: Bar graphs showing the methylation change in genes involved in circadian rhythm process in breast cancer. In tumor samples, the increase in the level of methylation in *DRD1*, *PTGDS*, *CASP1*, and *PGLYRP1* genes are observed.

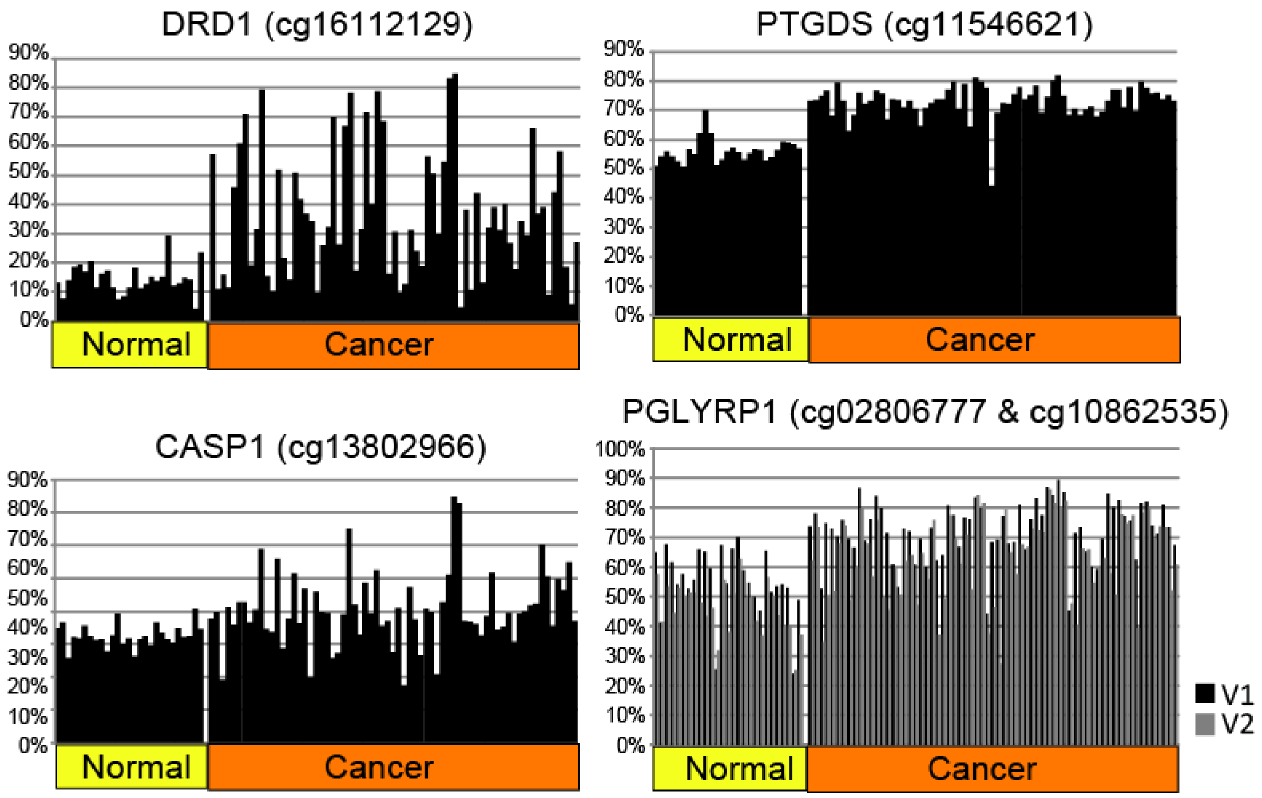

Supplement: Additional file 2 — Figure S1. Waterfall plots showing the methylation change in significant genes between normal and tumor samples involved in neurogenesis and epidermis development (GO terms). Positive values indicate hypermethylation in cancer, while negative values indicate hypomethylation in cancer. A. Neurogenesis. B. Epidermis Development. Figure S2. Change in average percent methylation of HOX gene family, PAX gene family, and WT1 involved in Transcription Factor Activity. Figure S3. Unsupervised clustering of probes involved in Sequence-specific Transcription Factor Activity. Figure S4. The status of PRC2 targets and CpG islands for those probes involved in the specified GO terms. Figure S5. The proportion of differentially methylated genes among the PRC2 targets and non-PRC2 targets (those probes with the p-value<0.05 and the minimum difference between the average methylation percentage of tumor vs. normal greater than 5% are graphed) A. Ectoderm Development. B. Epidermis Development. C. Embryo Development. D. Neurogenesis. Figure S6. Clustering of metabolite, drug target, and transcription factor concepts. Hypomethylated concepts are shown in red and hypermethylated concepts are shown in green. A. Metabolite concepts. B. Drug concepts. C. Transcription Factor concepts. Figure S7. Change in average percent methylation of the probes for TP73, CDKN1A, 1B, 1C, 2A and 2B, 2C, 2D, and APC. Figure S8. Cancer-specific enriched concepts in LRpath directional analysis. Biological concepts enriched with a significant p-value < 1e-4 in one tumor type are listed in the table below. In myeloma, kinase activities are enriched among hypermethylated genes, and muscle-related processes and components are enriched among hypomethylated genes. In breast cancer, several circadian processes are shown up to be enriched among hypomethylated genes. Figure S9. Bar graphs showing the methylation change in genes involved in circadian rhythm process in breast cancer. In tumor samples, the increase in the [file 1471-2164-13-526-S2.pdf]
